# HLA Class I Molecules as Immune Checkpoints for NK Cell Alloreactivity and Anti-Viral Immunity in Kidney Transplantation

**DOI:** 10.3389/fimmu.2021.680480

**Published:** 2021-07-06

**Authors:** Burcu Duygu, Timo I. Olieslagers, Mathijs Groeneweg, Christina E. M. Voorter, Lotte Wieten

**Affiliations:** ^1^ Department of Transplantation Immunology, Maastricht University Medical Center, Maastricht, Netherlands; ^2^ GROW, School for Oncology and Developmental Biology, Maastricht University, Maastricht, Netherlands

**Keywords:** NK cell, solid organ transplantation, KIR, NKG2A, HLA class I

## Abstract

Natural killer (NK) cells are innate lymphocytes that can kill diseased- or virally-infected cells, mediate antibody dependent cytotoxicity and produce type I immune-associated cytokines upon activation. NK cells also contribute to the allo-immune response upon kidney transplantation either by promoting allograft rejection through lysis of cells of the transplanted organ or by promoting alloreactive T cells. In addition, they protect against viral infections upon transplantation which may be especially relevant in patients receiving high dose immune suppression. NK cell activation is tightly regulated through the integrated balance of signaling *via* inhibitory- and activating receptors. HLA class I molecules are critical regulators of NK cell activation through the interaction with inhibitory- as well as activating NK cell receptors, hence, HLA molecules act as critical immune checkpoints for NK cells. In the current review, we evaluate how NK cell alloreactivity and anti-viral immunity are regulated by NK cell receptors belonging to the KIR family and interacting with classical HLA class I molecules, or by NKG2A/C and LILRB1/KIR2DL4 engaging non-classical HLA-E or -G. In addition, we provide an overview of the methods to determine genetic variation in these receptors and their HLA ligands.

## Introduction

Kidney transplantation is considered to be the best treatment option for patients with end-stage renal failure since transplantation gives better survival outcome and improved quality of life compared to dialysis (1). After transplantation, a primary concern is the function of the allograft, which can fail at early or late stage post transplantation due to various complications including allograft rejection and the occurrence of infections.

Allograft rejection is the result of concerted actions of several immune effector cells. During allograft rejection, the immune cells of the recipient get activated by alloantigens of the donor leading to immune responses against the graft and subsequently pathological changes, that can destroy the graft if not controlled (2). Rejection can occur minutes or days after transplantation (hyper acute rejection), weeks or months after transplantation (acute rejection) and months or years after transplantation (chronic rejection) (3). Although the advancements in pre-transplant immune monitoring and immunosuppression regimens lead to decreased incidence of hyper acute and acute rejection, chronic rejection remains a major hurdle of long term graft function (4). Therefore, novel treatment strategies need to be developed to improve the outcome after kidney transplantation. In order to achieve this, it is crucial to understand the major and critical players beyond B and T cells. Since Natural Killer (NK) cells can influence T-cell- or antibody-mediated allograft rejection (5) and have a central role in anti-viral immunity (6, 7), it would be highly relevant to further explore their role in kidney transplantation.

NK cells are innate lymphocytes that represent 5-15% of lymphocytes in peripheral blood, they are derived from common lymphoid progenitors, they can mediate both cytotoxic and cytokine producing effector functions, and they have been recognized primarily for their contribution to the immune response against intercellular pathogens and malignant cells (8). However, increasing evidence suggests a role for NK cells in allograft rejection after kidney transplantation as well (9–11). In contrast to T cells, NK cells do not require priming with an antigen and NK cell activation is regulated by the balance between inhibitory- and activating receptors. Engagement of inhibitory NK cell receptors with their cognate ligands will trigger an inhibitory signalling cascade in the NK cells, hence, setting the threshold for activation of the NK cells (12). Activating NK cell receptors, on the other hand, typically interact with ligands that are associated with cellular stress or with viral infection and that are highly expressed on virus-infected or malignant cells (13–15). An excess amount of activating receptor-ligand interaction will trigger NK cell activation even in the presence of low levels of inhibitory signaling, a condition called “induced-self” (16). NK cells will normally not attack healthy cells, since they do not, or only very lowly, express activating ligands (16).

MHC class I molecules are the most important inhibitory ligands for NK cells. Under normal circumstances, recognition of self MHC molecules by the inhibitory receptors prevents NK cell activation against host cells and creates self-tolerance (17). However, virally infected- or malignant cells frequently downregulate MHC class I molecules to escape from T cells and this decreased expression of HLA molecules lowers the threshold for NK activation making only a very small activating signal enough to trigger NK effector responses, a condition that is also known as ‘missing self’ (18). Simultaneously, the expression of ligands for activating receptors increases due to cellular stress, infection or tumorigeneses, and a shift from inhibitory signals to activating signals results in activation of NK cells, leading to elimination of target cells *via* NK cell mediated cytotoxicity or through secretion of pro-inflammatory cytokines (19).

First evidence for the role of NK cells in rejecting allografts originates from murine studies showing that F1 hybrids can reject parental bone marrow cells upon transplantation, so called “hybrid resistance” (20, 21). In the late 1980’s, the concept of NK cell “missing self recognition” was introduced after the observation that H-2 deficient lymphoma’s were rejected in a NK cell dependent manner (22). Missing self recognition implies that, in the absence of engagement of inhibitory NK receptors with “self MHC class” molecules (or H-2 in mice), NK cells more readily respond to foreign- or malignant cells lacking expression of those self MHC molecules. Hence, providing a direct link between F1 hybrid resistance and missing self recognition, and an explanation for NK cell allorecognition in bone marrow transplants. Traditionally, hybrid resistance has not been linked to solid organ transplantation and the Snell’s third law of transplantation states that ‘Grafts from either inbred parent strain to the F1 hybrid succeed’ (23). As will be discussed in more detail below, this idea was challenged by more recent studies in mouse models showing the participation of missing self-induced activated NK cells in cardiac allograft endothelial damage and vascular rejection, thereby, providing support for the existence of NK cell alloreactivity in solid organ transplantation when the endothelial cells are predominantly from donor origin (10, 24).

**Figure 1:**
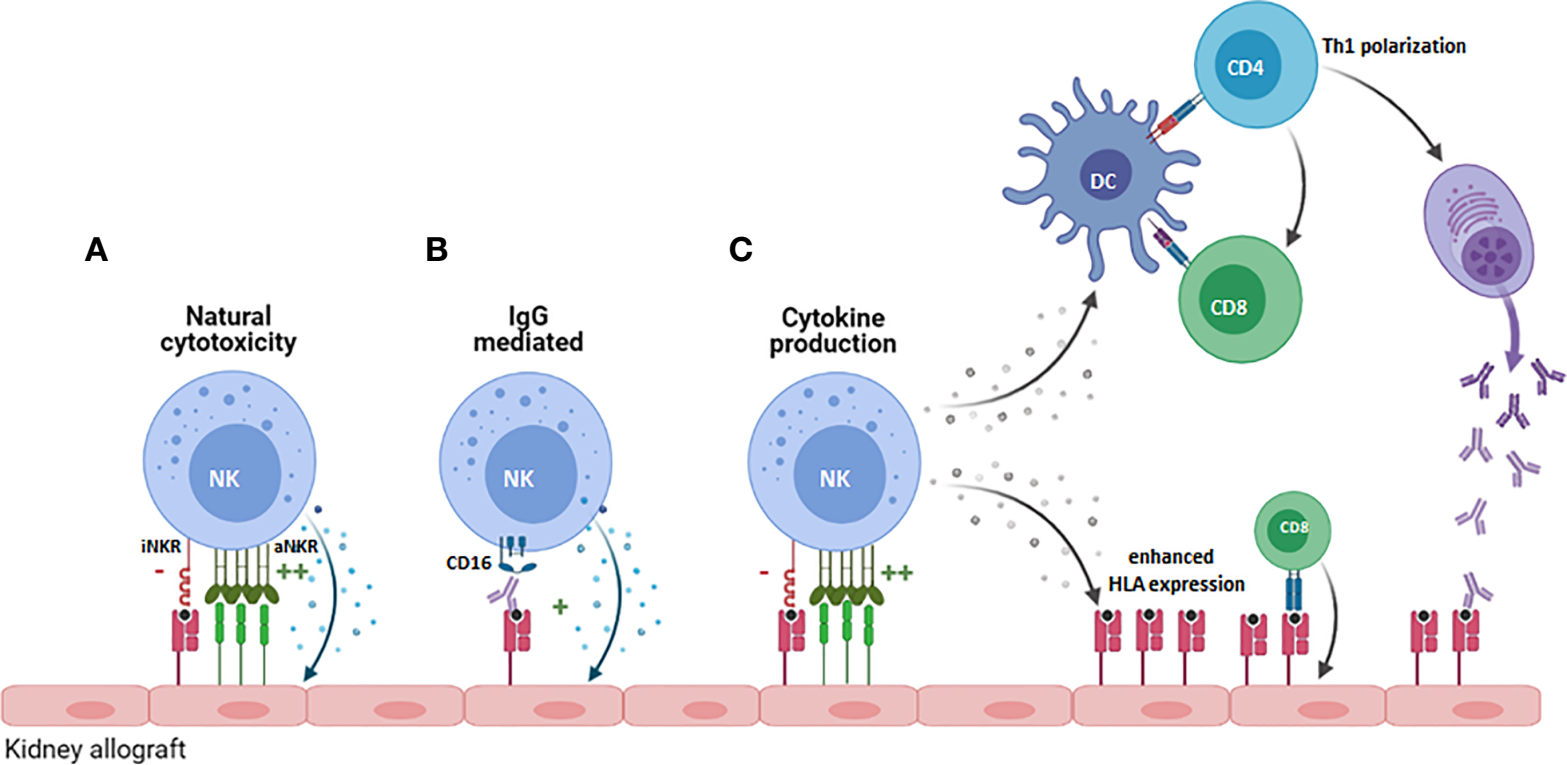
Potential role for NK cells in promoting allograft rejection. NK cells can contribute to allograft rejection in several ways: **(A)** By mediating direct cytoxicity against cells of the allograft that increasingly express cellular-stress or virus-associated activating ligands. **(B)** *Via* antibody-dependent cellular cytoxicity upon binding of CD16 on the NK cell to anti-HLA antibodies. **(C)** By producing proinflammatory cytokines like IFN-ϒ, that promote Th1 polarization of CD4^+^ cells, priming and activation of CD8^+^ T cells directed against the allograft and by stimulating B cell production of pathogenic IgG antibodies. iNKR, inhibitory NK cell receptor; aNKR, activating NK cell receptor.

Activated NK cells could influence allograft rejection in multiple ways (**Figure 1**): One way is *via* their influence on the adaptive arm of the immune system through their crosstalk with dendritic cells (DCs) (**Figure 1C**). The underlying mechanism is that DCs induce NK cell activation *via* secretion of cytokines such as type I IFN, IL-12 or TNFα (25–28), and that, as a result of this activation NK cells release TNFα and IFN-ϒ, which further promotes DC maturation (29, 30). Since DCs play a key role in T cell activation, enhanced maturation of DCs and NK-derived IFN-ϒ promote T cell activation, expansion, and Th1 polarization (31, 32). Infiltration of CD56^pos^ NK cells has been associated with poor outcome of kidney transplantation and interstitial fibrosis (33, 34). A more recent study, used flow cytometry to obtain more in depth profiles of NK cells revealing that CD56^bright^ NK cell infiltrates were enhanced in biopsies from patients experiencing T cell mediated rejection (TCMR) while CD56^dim^ NK cell infiltrates characterized biopsies from patients with antibody mediated rejection (AMR) (35). Since CD56^bright^ NK cells are potent producers of cytokines, this was suggestive of a role for CD56^bright^ NK cells in the recruitment and activation of alloreactive T cells (35). Moreover, this may be enhanced by the production of IFNϒ by activated T- or NK cells contributing to inflammation-induced enhanced expression of HLA alloantigens (35). Infiltration of CD56^dim^CD16^pos^ NK cells in AMR, would fit with the direct impact that NK cells can have on antibody-mediated allograft rejection *via* the Fc receptor CD16 expressed on the NK cell surface (**Figure 1B**) (36, 37). CD16, or FcϒRIIIa, can bind to the Fc part of donor specific antibodies (DSA), generated by the recipient B cells, that bind to the HLA or non-HLA molecules present on the allograft endothelial cell surface thereby inducing antibody dependent cellular cytotoxicity (ADCC) (38). In a mouse model for kidney transplantation, high DSA titres were paralleled with infiltration and proliferation of NK cells in the allograft, and in the absence of NK cells, the DSA could not provoke acute AMR and triggered progressive chronic kidney injury and the enhanced expression of pro-fibrotic genes leading to failure of kidney function (39). In humans, NK cells have been shown to contribute to chronic AMR by participating in DSA-induced microvascular inflammation (MVI) (33, 40). Consequently, NK cells can significantly contribute to the damaging effects that DSA can have on graft endothelial cells and by mediating DSA-induced ADCC they can also worsen the outcome of complement-independent chronic AMR (40).

In addition to producing cytokines and mediating ADCC, NK cells can directly damage donor endothelial cells that overexpress activating NK cell ligands (**Figure 1A**) or lack inhibitory HLA ligands for the patients NK cells as a result of mismatched HLA class I molecules between donor and recipient cells, a concept called “missing-self recognition” (10). The functional relevance of this type of response was elegantly demonstrated in a study showing chronic vascular rejection in approximately half of the DSA negative kidney transplant patients (10). In subsequent *in vitro* and *in vivo* models, missing-self by itself was not sufficient for the NK cells to damage graft endothelial cells. However, in combination with Poly-I:C (as surrogate for viral infection) or ischemia/reperfusion as provoking factor for NK cell activation and expression of activating ligands on endothelial cells, microvascular inflammatory lesions and allograft rejection occurred in an NK cell dependent manner (10). While chronic AMR is difficult to treat with existing immunosuppressive agents, mTOR inhibitors were effective in reducing NK-mediated rejection in this preclinical model (10), illustrating that better understanding of the role of NK cells in solid organ transplantation and how they synergize with the adaptive immune system could help to develop improved personalized immunosuppressive therapies.

The difficulty with studying NK cells in the kidney transplantation setting is that they act as a double edged sword. In addition to the above mentioned role in promoting acute- and chronic allograft rejection, a role for NK cells in inducing transplant tolerance has been proposed by several groups [reviewed in (41)]. One of the primary reasons is that NK cells in the stem cell transplantation setting have been shown to kill immature DCs and by doing so they prevent activation of alloreactive T cells and graft versus host disease (42). Comparable observations have been made in skin transplantation models (43). Moreover, NK cells have been shown to be able to kill highly activated T cells and to induce regulatory T cells which would also contribute to tolerance (41).

Besides allograft rejection, infection is an important contributor to the morbidity and mortality after kidney transplantation (44). Kidney transplant recipients receive immunosuppressive therapy in order to prevent rejection and to maintain allograft function, however, this therapy makes the transplant patients predisposed to various infections including viral infections. NK cells are critical players in the antiviral immune response and their contribution in controlling viral infections is a second reason why NK cell activation could be very beneficial in the transplant setting. The most frequent viral infections post transplantation occur mainly with viruses such as cytomegalovirus (CMV), Herpes simplex (HSV), Epstein Barr Virus (EBV), BK polyomavirus, Hepatitis B and C (44). These infections can drive from primary infection, transmission from the donor and reactivation of latent infections. Their effect on transplantation outcome emerges *via* different mechanisms; infection can either lead to an invasive disease such as CMV disease seen upon CMV reactivation in immunosuppressed individuals (45) or can contribute to graft rejection indirectly (46, 47). As a response to infection, virus specific CD8+ T cells can be formed, which can show cross reactivity with donor alloantigens (also known as ‘molecular mimicry) inducing alloreactive response against donor cells and eventually leading to rejection (48). In a healthy individual, CD8+ T cells and NK cells are primary responders and controllers of viral infection. Even more, NK cells have been reported to keep the CMV infection under control in the absence of CD8+ T cells in a child with severe combined immunodeficiency syndrome (49). Both CD8 T cells and NK cells kill virally infected cells by the release of granules containing perforins and granzymes leading to a reduction in membrane integrity and the activation of apoptosis promoting caspase activity and resulting in lysis of the target cell (50). Activation of CD8 T cells and NK cells is very different: CD8 effector T cells get activated upon interaction with a cell presenting viral peptides on MHC class I, while NK cells require the expression of virally-induced ligands for receptors like NKp30 and NKp46 (16). Since MHC class I acts as an inhibitory ligand for NK cells, NK cells are especially useful in eliminating target cells that reduced MHC class I in an attempt to escape from CD8 T cell immunity (17).

In kidney transplant recipients, it has been shown that reduced NK cell function correlates with and is a predictor of severe infection indicating the requirement of intact NK cell function in the defence against viruses (49, 51). Besides, the number of activating NK cell receptor genes of the Killer Immunoglobulin-like receptor (KIR) family in the recipients have been associated with decreased incidence of Human Cytomegalovirus (HCMV) *de novo* infection and reactivation in the first year of kidney transplantation (52). Moreover, the KIR gene repertoire can determine susceptibility of the patients to HCMV infections and the severity of the infection in renal transplant patients (53). Although the phenotypic and functional profile of NK cells has been reported to be altered under the immunosuppression treatment (54), their recovery has been detected to be faster than T cells (55). All these findings indicate the contribution and significance of NK cells in response to anti-viral defence, even in the absence of T cell response, in kidney transplantation.

The biology of NK cells, the multiple roles NK cells can have in organ transplantation and a detailed analysis of the interplay between viruses and NK cells have been excellently reviewed before, for example in (5, 41). For an overview on these topics we refer to these previous reviews. In the present review, we will primarily focus on the impact of HLA class I on NK cell alloreactivity and anti-viral immunity in the kidney transplantation setting and how this may impact transplantation outcome. This is an important topic because, although NK cells can respond to a plethora of ligands, classical and non-classical HLA class I molecules represent the most important inhibitory immune checkpoints for NK cells. As will be discussed in more detail in this review, the magnitude of the effect of HLA can depend on the degree of matching between donor and recipient and on the local microenvironment in the graft. Moreover, there are different models describing how the interaction of HLA molecules and NK cell receptors can affect the outcome in kidney transplantation. In the current review, we aim to describe those models and highlight recent findings regarding the HLA class I molecules as immune checkpoints in the regulation of NK cell alloreactivity in kidney transplantation as well as their influence on NK cell responses in viral disease occurring upon transplantation. In addition we will provide an overview of the state of the art methods used to identify NK cell receptors and HLA molecules and we discuss their limitations.

## Classical HLA Class I Molecules as Ligands for Inhibitory- and Activating NK Cell Receptors

The highly polymorphic classical HLA class I molecules HLA-A, HLA-B and HLA-C are critical regulators of NK cell activation. These molecules are expressed by virtually every healthy cell and they present peptides derived from intracellular proteins on their cell surface and by doing so they play a critical role in discrimination between self- *vs* non-self- or diseased cells (56). Classical HLA class I molecules can interact with inhibitory- as well as activating NK cell receptors of the KIR family. Moreover, during NK cell functional maturation, their interaction with inhibitory KIR (iKIR) family members results in licensing of NK cells and licensed NK cells can more potently respond upon activation by a potential target cell than their unlicensed counterparts (19, 57). Licensing -or ‘NK cell education’- is the process where NK cells interact *via* iKIR with classical HLA class I molecules expressed on for example stromal cells in the bone marrow, and licensed NK cells are characterized by an increased density in cytotoxic granules as well as a slightly altered metabolism with enhanced levels of glycolysis (58). To avoid excessive activation of NK cells against normal healthy cells, HLA class I molecules also act as potent inhibitory ligands and by doing so, they set the threshold for NK cell activation (59).

**Figure 2:**
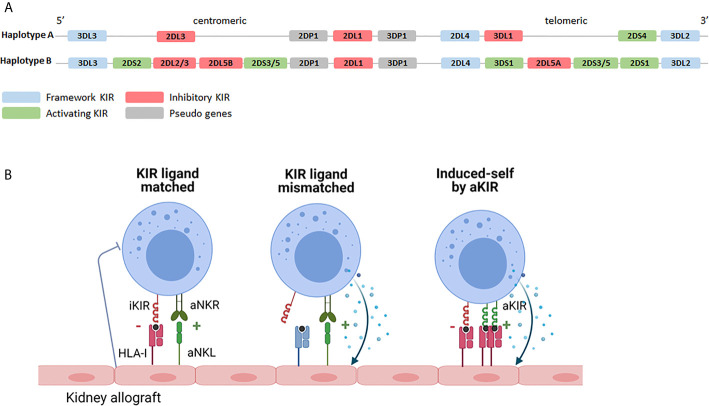
KIR haplotypes and the potential effects of KIR-HLA interaction. **(A)** Based on the *KIR* gene content two haplotypes can be distinguished. The A haplotype containing *KIR2DS4* as the only activating receptor and the B haplotype containing multiple combinations of activating- and inhibitory *KIR* genes. Depending on the exact combination of *KIR* genes, multiple different B haplotypes are known (https://www.ebi.ac.uk/ipd/kir/sequenced_haplotypes.html). **(B)** Recipient NK cells may encounter their HLA class I ligands on the kidney allograft (KIR ligand match) or not (KIR ligand mismatch). Even in the presence of class I ligand, stress- or infection associated ligands for activating receptors, including activating KIR, on the allograft can trigger NK cell cytotoxicity (induced-self by aKIR). iKIR, inhibitory killer immunoglobulin-like receptor; aKIR, activating killer immunoglobulin-like receptor.

NK cells interact with classical HLA *via* so called KIR receptors, encoded on chromosome 19. The KIR family comprises several inhibitory- and activating family members and they are named by the presence of two (KIR2D) or three (KIR3D) extracellular immunoglobulin domains. Activating family members (aKIR) have a short (KIRxDSx) intracellular domain with immunoreceptor tyrosine-based activation motifs (ITAMs) and, although some of them recognize HLA class I, for several of the aKIRs the ligands remain elusive. Inhibitory family members have a long (KIRxDLx) intracellular domain with immunoreceptor tyrosine-based inhibitory motifs (ITIM) and most of them have HLA class I as ligands (60). Like their HLA ligands, KIRs are highly polymorphic and differences in expression levels due to copy number variation and allelic variation (including several known null-alleles) have been described (61). NK cells acquire KIR during maturation in a stochastic manner and can express none, one or a combination of KIRs, leading to high variation in expressed KIR repertoires between individuals as well as between NK cells within an individual (62). Moreover, depending on the exact set of *KIR*-genes, multiple haplotypes are known (https://www.ebi.ac.uk/ipd/kir/sequenced_haplotypes.html). The so called framework KIRs (*KIR3DL3, KIR3DP1, KIR2DL4* and *KIR3DL2*) are present in every haplotype (63). Furthermore, the haplotypes can be distributed in haplotype A or haplotype B (**Figure 2A**). The A haplotype consists of *KIR2DL1, KIR2DL3, KIR2DL4, KIR3DL1, KIR3DL2, KIR3DL3, KIR2DP1* and *KIR3DP1* and only *KIR2DS4* as activating receptor (63). The set of *KIR* genes present in the B haplotype is much more diverse and multiple aKIRs are typically expressed (63). As will be discussed in more detail later, the introduction of next generation sequencing (NGS) led to a continuous increase in the number of *KIR* alleles and haplotypes.

## Impact of KIR-HLA Class I Interaction on Kidney Transplantation Outcome

KIR-ligand interaction may influence transplantation outcome on the one hand by promoting NK cell activation through the interaction between activating family members and their HLA ligands or by dampening NK cell activation *via* inhibitory family members. The influence of iKIRs is best described for KIR2DL1, binding to HLA-C alleles harboring the C2 epitope (lysine at AA position 80); for KIR2DL2/3 that predominantly interact with HLA-C alleles with the C1 epitope (asparagine at AA position 80); and for KIR3DL1 binding to HLA-B alleles with a Bw4 epitope and to HLA-A*23/*24/*32 (64, 65). Since many kidney transplantations are performed with one or more mismatches in HLA-A, -B or -C, iKIR receptors expressed on the patients NK cells may or may not encounter their HLA class I ligands on cells of the transplanted kidney, a so called “KIR-ligand match” or “KIR-ligand mismatch” (**Figure 2B**). The functional relevance of a KIR-ligand mismatch is that the amount of inhibitory signals provided to the NK cells is reduced making the NK cells more prone to attack a potential target cell. In a landmark paper by Ruggeri et al., patients that received a haploidentical stem cell graft that was iKIR-ligand mismatched in the graft *vs.* host direction had less relapse of disease which could be attributed to a better anti-tumor response of graft NK cells (42). In addition, these patients developed less graft-versus-host disease than patients receiving a KIR-ligand matched stem cell graft which could be explained by the enhanced killing of host immature dendritic cells by graft NK cells in iKIR-ligand mismatched receivers (42). Given the relatively short lifespan of NK cells and because of the lack of massive clonal expansion by NK cells, stable long term presence of donor NK cells seems rather unlikely in the kidney transplantation setting. Nevertheless, the presence of passenger donor NK cells, transferred from the donor to the recipient in the kidney allograft, has been shown at the time of transplantation (66). These donor NK cells have been suggested to get activated upon ischemia/reperfusion damage and *in vitro* studies showed that these activated NK cells can subsequently promote maturation of DC hence contributing to enhanced allorecognition by T cells (66). The role of recipient NK cells has been studied much more extensively and will be discussed in the following paragraph in more detail.

In kidney transplantation, recipient NK cells can contribute to graft rejection or antiviral immunity by mediating natural cytotoxicity, triggered upon encountering a target cell expressing high levels of stress- or infection- associated ligands for activating receptors (**Figures 1A**, **2B**). Alternatively they can contribute to AMR after engagement of the CD16 Fc receptor on the NK cell and an antibody bound to the cells of the allograft (**Figure 1B**). Furthermore, upon activation, they can produce IFN-ϒ and by doing so act as adjuvants for the adaptive immune system by promoting Th1 and CD8^+^ T cell activation (**Figure 1C**). iKIR licensed NK cells have been shown to have higher density granules than non-licensed cells enabling them to more potently mediate all these effector functions (58). Simultaneously, NK cells expressing iKIRs that encounter their cognate HLA ligand on the allograft will have a higher activation threshold than mismatched NK cells that do not meet their HLA ligand, making it relevant to address KIR- and HLA status in the kidney transplantation setting. The impact of KIR-ligand matching *vs* mismatching in HvG direction on graft rejection and/or graft survival has been addressed in multiple studies (see **Table 1**). In a cohort of 69 patients, Kreijveld et al. observed no association between the occurrence of acute rejection after reduction of immune suppression and KIR-ligand matching status, presence of KIR-ligands in the donor or NK cell frequencies or subsets (67). In line with those data, no impact of KIR-ligand matching status on long term allograft function was found in cohorts of 126 (68), or 2757 (69) renal transplant patients, the first one with 5 years follow up, the latter with 10 years. In another study, patients with stable renal function (n=119) were compared with patients experiencing acute rejection within the first 3 postoperative months (n=105), demonstrating that HLA-C ligand compatibility by itself had no influence on transplantation outcome while a higher number of inhibitory recipient *KIR* genes, a higher number of KIR2DL2/DS2 matches and a higher number of mismatches for KIR2DL3 were detected in the non-rejectors (70). Higher number of inhibiting KIR genes encountering their ligand might hinder NK cell activation and thus lead to less damage to the kidney. Indeed this is in accordance with the results of the study in which the absence of recipient KIR2DL1-donor HLA-C2 and/or recipient KIR3DL1-donor-HLA-Bw4 was significantly higher in patients experiencing chronic rejection (71), with the background that these combinations have higher NK inhibitory capacity than the combination KIR2DL2/3 with HLA-C1. In two studies (72, 73) the authors investigated the effect of KIR-ligand mismatches on long term graft survival in a cohort of patients transplanted with a HLA-A, -B, -DR compatible donor, with the idea that in this cohort the NK cell effects cannot be obscured by allo-reactive T and/or B cells. Indeed, in one study with 137 patients with HLA-A, -B, -DR compatible donors, KIR-ligand mismatches were associated with a 25% reduction in 10 year graft survival, whereas no effect was seen in 260 patients with a HLA-A, -B incompatible, HLA-DR compatible transplantation (72). However, in the other study no effect of KIR-ligand mismatches was observed in 608 patients with a HLA-A, -B, -DR compatible transplantation (73). These contradictory results were rather striking, because the groups seemed to be comparable. Whether this contradiction is due to the difference in numbers of patients is unclear. In one paper only deceased donors were included with HLA-A, -B and -DRB1 zero mismatch with the patient (73), whereas in the other paper both deceased and living donors were included that were HLA-A, -B, -DR compatible, which might have included donors matched for HLA-A and –B at the broad level instead of at the split level (72). Interestingly, the difference in graft survival between KIR-ligand match and mismatch appeared after 5 years posttransplantation, so no difference was observed in 5 years survival curves (72). Koenig et al. challenged the idea that antibodies are the only primary trigger for microvascular inflammation in kidney transplantation (10). In a cohort of 129 patients without DSA, and in *in vitro* and *in vivo* models, they showed that in approximately half of patients MVI lesions were not mediated by antibodies but by activation of innate immune cells and that the presence of a KIR-ligand mismatch in HvG direction enhanced this process (10). In a mouse model they also demonstrated that NK cell activation in response to KIR-ligand mismatched microvascular endothelial cells was mTORC1 dependent and inhibition of mTORC1 with rapamycin could prevent this type of rejection (10). In a recent follow up study with 1682 kidney transplant patients, the same authors demonstrated that KIR-ligand mismatches have an increased detrimental effect on transplantation outcome in patients with non-complement fixing DSA as they promote AMR mediated by DSA that trigger NK cell reactivity against graft endothelial cells (40). Also these two studies support the hypothesis that KIR-ligand incompatibility has a detrimental effect on allograft survival even in a situation with DSA.

**Table 1:** Overview of published studies investigating the impact of KIR-ligand matching and mismatching on graft survival and graft rejection after kidney transplantation.

Patient Group	n	Cases	n	Controls	n	Outcome/Variable	Observations	Reference
reduced immune suppression	69	acute rejection	24	no acute rejection	45	peripheral blood NK cell frequency	no differences between case and control	Kreijveld et al. (67)
						presence of single KIR genes in recipient	no differences between case and control	
						presence of KIR haplotypes in recipient	no differences between case and control	
						presence of NK cell alloreactivity based on missing self	no differences between case and control	
						presence of NK cell alloreactivity based on missing ligand	no differences between case and control	
deceased donors	126	WGF (5year eGFR/creatinine)	59	SGF (5 year eGFR/creatinine)	67	NK alloreactivity (recipient KIR/HLA donor mismatch ligand)	no differences between case and control	La Manna et al. (68)
deceases donors	2757	KIR ligand incompatible	871	C1/2-Bw4matched	1416	graft survivalrate10 year follow up	no differences between case and control	Tran et al. (69)
		KIR ligand incompatible	871	C1/2-Bw4mismatched	470	graft survivalrate10 year follow up	no differences between case and control	
unrelated donors	224	with AR within 3months	105	With stable renal function	119	HLA·C ligand incompatibility	no differences between case and control	Kunert et al. (70)
						Donors homozygous for C2	higher in controls compared to cases	
						number of recipient inhibitory receptors	higher in controls compared to cases	
						number of donor ligand matches for recipient KIR2Dl2/DS2	higher in controls compared to cases	
						number of donor ligand mismatches for recipient KIR2Dl3	higher in controls compared to cases	
HLA-DR matched deceased	174	chronic rejection	42	SGF	132	Donors homozygous for C1	higher incases compared to control	Littera et al. (71)
donors						absence of rKIR2Dl1/dHLA-C2	higher incases compared to controls	
						absence of rKIR3DL1/dHLA-Bw4	higher incases compared to controls	
HLA·AB incompatible, DR compatible donors	260	KIR ligand mismatched	134	KIR ligand matched	126	10year graft survival	no differences between case and control	Van Bergen et al. (72)
HLA ABDR compatible donors	137	KIR ligand mismatched	42	KIR ligand matched	95	10year graft survival	25% reduction in graft survival cases of controls	
HLA-ABDR compatible, deceased donors	608	KIR ligand mismatched	193	KIR ligand matched	415	10year graft survival	no differences between case and control	Tran et al. (73)
Kidney transplant patients	760	C2present	457	C2 absent	303	long term graft survival	shorter in cases compared to controls	Hanvesakul et al. (66)
						acute rejection	no significant differences between case and control	
Kidney allograft biopsies: MVI+DSA+, non-complement	62	missing-self present	21	no missing-self present	23	graft survival	lower incases compared to controls	Koenig et al. (40)
Kidney allograft biopsies: MVI+, DSA+, complement	73	missing-self present	23	no missing-self present	17	graft survival	no difference between cases and controls	

WGF, worse graft function; SGF, stable graft function; AR, acute rejection.

Evaluation of the impact of KIR-ligand matching status, and comparison between clinical studies is complicated by multiple factors: First of all the differences in study design and clinical parameters like the exact transplantation protocol, the presence or absence of preexisting or *de novo* DSA, the outcome parameters used for evaluation and immunosuppressive regimens maybe different between studies. A second important factor is the definition of KIR-ligand incompatibility. Ideally the phenotypic presence of KIR receptors should be confirmed. This is important because the genes encoding iKIRs are not always present in every individual and for e.g. KIR2DL1 and KIR3DL1 null alleles exist (https://www.ebi.ac.uk/ipd/kir). When assessing the presence *vs* absence of the HLA ligands it is especially for Bw4 important to also consider the HLA-A alleles as HLA-A*23, -*24 and -*32 encode for the binding site for KIR3DL1 and this is not always consistently done between studies. Since NK cell can express one or more KIRs, and because there is a large inter-individual variation, the size of the alloreactive NK cell population (i.e. the size of the population of NK cells expressing only mismatched KIRs) can be very different on a per individual basis (62). Especially in smaller studies this may influence the overall impact of KIR-ligand mismatching. Thirdly, the microenvironment in the graft or the systemic inflammatory status of the patient may influence the impact of KIR-ligand incompatibility. Under homeostatic conditions, even in the situation with a complete lack of inhibitory signaling *via* HLA and KIR, unactivated NK cells will not attack healthy cells. The absence of inhibitory signaling is not enough to activate the NK cell and NK activation requires expression of activating ligands on a potential target cell (14). Viruses frequently encode activating NK ligands, and cellular stress or proinflammatory cytokines promote activation of NK cells by enhancing expression of activating ligands on potential target cells or by increasing expression of activating receptors on NK cells (15). This makes viral status and factors like ischemia/reperfusion important influencers of the NK cell response in kidney transplantation.

NK cells are critical controllers of viral infections, this may be especially relevant in conditions were T cell mediated control is reduced as a result of immunosuppression regimes. HCMV infection is one of the most frequently occurring complications after kidney transplantation, a risk factor for the rate of graft loss and associated with reduced survival (74). Several studies confirmed the functional relevance of the *KIR* gene repertoire for HCMV infection upon kidney transplantation (see **Table 2**). Most pronounced are the associations observed for the presence of KIR haplotype B/X, especially the telomeric B haplotype, and reduced HCMV infection or reactivation (52, 75–77, 82), which presumably could be explained by the presence of a higher number of activating KIR’s in the KIR B/X-haplotype as compared to the KIR AA-haplotype, facilitating NK cell mediated killing of virally infected cells. One study, however, describes the opposite effect in two independent cohorts, but both consisting of patients that were HCMV negative at the time of transplantation, but transplanted with an HCMV positive donor (53). In both cohorts the KIR telomeric haplotype B/X in combination with HLA-C2 was significantly associated with susceptibility to HCMV infection, whereas the KIR haplotype AA in combination with HLA-C1 was protective for development of severe disease (53). In another study the significant effect of the KIR B haplotype as protection for HCMV infection, was only detected in patients that were already HCMV seropositive at the time of transplantation (77). A third study with 90 kidney patients that were HCMV negative and transplanted with a HCMV positive donor showed a trend towards a lower incidence of HCMV infection in recipients with KIR AA haplotype (81). It is not clear which biological mechanism is underlying these findings for HCMV seronegative patients at the time of transplantation and whether the lack of T or NK cell memory in this specific group in combination with immunosuppression of T cell responses during the initial stage of infection plays a role. A protective role for KIR B haplotypes has also been observed for Varicella zoster (78) and BK virus (79) though two other studies did not find a significant impact of KIR haplotypes on BK virus (78, 80). The impact of KIR repertoires on other viral infections is not very well studied.

**Table 2:** Overview of published studies investigating the impact of KIR and KRI-ligand on viral infections after solid organ transplantation.

Patient Group	n	Cases	n	Controls	n	Outcome/Variable	Observation	Reference
kidney transplant patients	122	KIR haplotype B/X	82	KIR haplotype AA	40	rate of CMV infection 1st year after tx	significantly lower in cases (20%) compared to controls (36%)	Stern et al. (52)
						graft function	no significant differences between case and control	
						rate rejection episodes	no significant differences between case and control	
						rate of EBV, BKV, Herpes simplex	no significant differences between case and control	
kidney cohort 1:HCMVD+R- patients with antiviral prophylaxis	76	>500 copies HCMV/ml within first 6months	24	<500 copies HCMV/ml	52	frequency of Tel B KIR genes	higher in cases compared to controls	Jones et al. (53)
						Freq Tel B +HIA-C2	higher in cases compared to controls	
kidney cohort 2: HCMV D+R- patients without antiviral prophylaxis	65	HCMV >50 infected cells	12	HCMV<10 infected cells	35	Tel AA haplotype	significantly lower incases compared to controls	
						Tel A/X +HIA-C1	significantly lower in cases compared to controls	
						Tel B/X haplotype	significantly higher in cases compared to controls	
						Tel B/X +HLA-C2	significantly higher in cases compared to controls	
						Homozygous HLA-C2	significantly higher in cases compared to controls	
kidney transplant patients	196	two missing KIR ligands	38	no or 1 missing KIR ligand	158	rate of CMV infection up to 3 months	significantly lower in cases compared to controls	Hadaya et al. (75)
		HLA-C missing KIR ligand	103	no HLA-C missing KIR ligand	93	rate of CMV infection up to 3 months	significantly lower in cases compared to controls	
		patients with more activating KIR genes		patients with fewer activating KIR genes		rate of CMV infection up to 12 months	each additional activating KIR gene reduced risk of CMV event by 19%	
kidney transplant patients	339	patients with KIR Cen BX haplotype	192	patients with KIR Cen AA haplotype	147	rate of CMV infection up to 12 months	no significant differences between case and control	Stern et al. (76)
		patients with KIR Tel BX haplotype	158	patients with KIR Tel AA haplotype	181	rate of CMV infection up to 12 months	significantly lower in cases compared to controls	
kidney patients excluding D-R-	223	patients with KIR B/X haplotype		patients with KIR AA haplotype		cumulative incidence of CMV in first 2 years	no significant differences between case and control	Gonzalez et al. (77)
kidney patients excluding D-R, receiving ATG	40	patients wtih KIR B/X haplotype		patients with KIR AA haplotype		cumulative incidence of CMV in first year	38% incases *vs* 48% in controls	
heart, kidney, liver, lung tx patients	649	patients with KIR B/X haplotype	473	patients with KIR AA haplotype	176	cumulative incidence of varicella zoster infection (n=28)	significantly lower in cases compared to controls	Schmied et al. (78)
		patients with KIR B/X haplotype	473	patients with KIR AA haplotype	176	Cumulative incidence of EBV, HSV, BKPyV	no significant differences between case and control	
kidney transplant patients	158	patients with severe BKV reactivation	48	patients with no BKV and stable function	110	Tel B/X haplotype	significantly lower incases compared to controls	Trydzenskaya et al. (79)
				first 6 years after transplant		Low number of activating KIR genes (<4)	significantly higher percentage incases compared to controls	
						presence of KRI3DSl	significantly higher in controls compared to cases	
						KIR/HLA match and mismatch	no significant differences between case and control	
kidney transplant patients	103	patients with KIR B/X haplotype	75	patients with KIR AA haplotype	28	cumulative incidence of BK virus in first 2 years	no significant differences between case and control	Brochot et al. (80)
kidney transplant patients D+R-	90	patients with KIR B/X haplotype	63	patients with KIR AA haplotype	27	cumulative incidence of CMV in first year	trend towards lower incidence in controls (30%) *vs* cases (48%)	Michelo et al. (81)
		one or more missing KIR ligands	38	no missing KIR ligand	52	cumulative incidence of CMV in first year	no significant differences between case and control	
kidney transplant patients	138	patients with KIR B/X haplotype	96	patients with KIR AA haplotype	42	cumulative incidence of CMV in first 2 years	trend toward slower incidence in cases (31.2%) *vs* controls (47.6%)	Deborska-Materkowska et al. (82)
		CMV infection	50	no CMV infection	88	lack of KIR2DS2	significantly higher in cases compared to controls	
		CMV infection	50	no CMV infection	88	presence of KIR2DL3	significantly higher in cases compared to controls	
		CMV infection	50	no CMV infection	88	presence of KRI2DL2-HLA-C1	significantly higher in cases compared to controls	

D+, HCMV positive donor; R-, HCMV negative recipient; D-, HCMV negative donor; MVI, microvascular inflammation; DSA, donor specific antibodies.

The relevance of inhibitory KIRs and of KIR-ligand incompatibility on control of viral infections is less conclusive and less well explored. In a first study, the absence of HLA-C ligands for the recipients inhibitory KIRs associated with reduced HCMV infection rate after transplantation (75). While a second study did not observe differences in *KIR* gene and genotype distribution and no effect of KIR-ligand mismatching for patients with or without HCMV (81). Also individual KIRs have been related to HCMV infection. In a study with 138 kidney transplantation recipients, the lack of KIR2DS2, the presence of KIR2DL3 or the combination of KIR2DL2 and HLA-C1 were identified as risk factors for HCMV infection (82). Moreover, enhanced numbers of KIR3DL1 positive NK cells have been observed early upon HCMV reactivation, and in an *in vitro* study, the authors subsequently demonstrate that this subset most efficiently kills HCMV infected fibroblasts (83).

The multiple studies showing associations between KIR haplotypes and kidney transplantation outcome illustrate the relevance of further exploring the functional consequences of KIR repertoires in combination with analysis of KIR ligands, in kidney transplantation outcome. In the following paragraphs we will discuss the different methods that can be used to determine KIR and HLA genotypes enabling such future studies.

## Methods to Determine the Presence of HLA Class I KIR Ligands

KIR ligands are defined by single amino acid differences on HLA-B and -C molecules. The HLA-B molecules can be divided into two supertypic specificities, Bw4 and Bw6, with amino acid differences at positions 77 and 80-83 of the mature protein. Bw6 is defined by serine at residue 77 and asparagine at residue 80, whereas Bw4 is characterized by at least seven different patterns of amino acids at positions 77 and 80-83. Complicating factor is that the Bw4 motif is also present on several HLA-A molecules. HLA-B and HLA-A (A*23, *24, *32) alleles carrying the HLA-Bw4 epitopes are recognized by KIR3DL1, the Bw6 epitope is not a ligand for KIRs. The HLA-C molecules carry either a C1 or a C2 motif, based on a dimorphism at residue 80. The C1 motif is defined by the presence of an asparagine at position 80, whereas the C2 motif has a lysine at position 80 of the mature protein (84).

**Figure 3:**
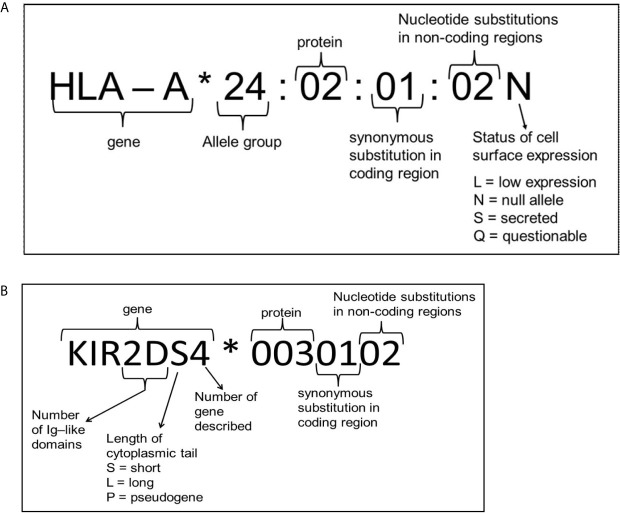
Explanation of nomenclature used for HLA **(A)** and KIR **(B)**. Figure adapted from the IPD-IMGT/HLA and IPD-KIR database website, respectively (85, 86).

**Figure 4:**
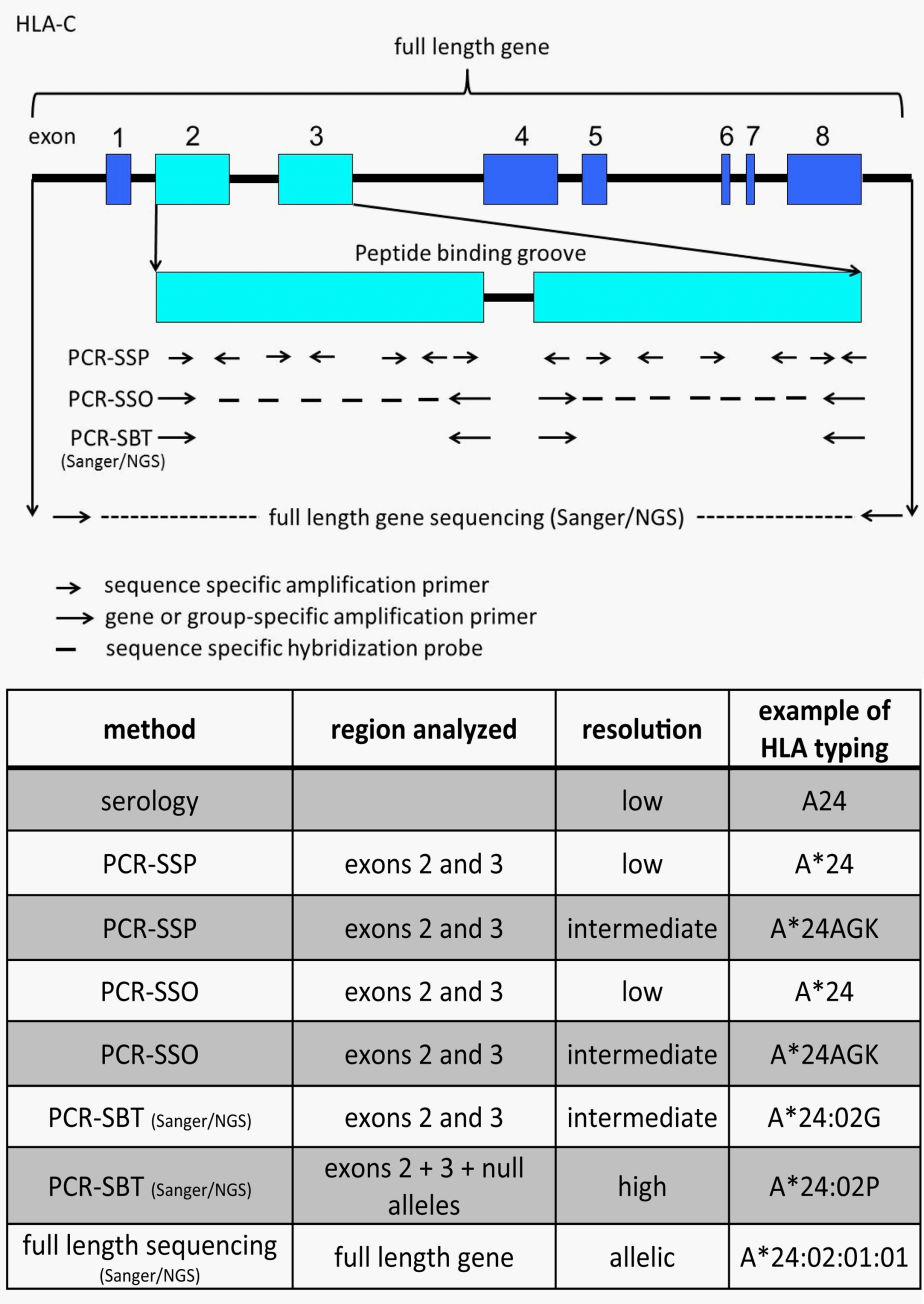
Illustration and comparison of different HLA typing methods and the generally obtained typing resolution level. The figure depicts HLA-C as an example. Depending on the number of sequence specific primers or probes the PCR-SSP/SSO method can have low or intermediate resolution typing result. A*24AGK: string of different A*24 alleles. A*24:02G: group of alleles with identical peptide binding groove, but differences outside (including null alleles). A*24:02P: group of proteins with identical peptide binding groove, but differences outside (excluding null alleles).

To determine these amino acid differences several different methods are available. HLA typing has started with serological determination, discriminating HLA molecules by incubating cells with known HLA antibodies using the complement dependent cytotoxicity (CDC) method. Since Bw4 and Bw6 are potent public antibody epitopes, specific antibodies against these supratypes are present and can be used to distinguish these motifs. However, nowadays HLA typing is merely determined by molecular typing methods using genomic DNA. Due to the high polymorphism a special HLA allele nomenclature has been developed for molecular HLA typing results, an example and explanation of this nomenclature is depicted in **Figure 3**. Molecular HLA typing can be performed at low or high resolution level, determining respectively the allele groups (comparable to serological types) or the alleles present, illustrated in **Figure 4**. Although most alleles within an allele group bear the same NK ligand motif, there are some exceptions as described previously (87) and as can be deduced from the protein sequences of the alleles available at the IPD-IMGT/HLA database (https://www.ebi.ac.uk/ipd/imgt/hla) (85). Therefore, low resolution HLA typing will not be sufficient to determine the NK ligands with certainty. In contrast, high resolution typing will determine the protein sequences of the peptide binding groove, encoded by exons 2 and 3 of the HLA class I gene, as a minimum, which is sufficient for defining the NK binding region.

For high resolution typing, sequencing is the most reliable method, because then the complete nucleotide sequence of at least exons 2 and 3 is determined. Other methods are PCR-SSP, based on amplification of genomic DNA with sequence specific primers analyzed by gel electrophoresis, and PCR-SSO, based on amplification of the locus of interest followed by hybridization with sequence specific oligonucleotide probes. Although typing can be performed with these methods, the problem exists that not all nucleotides are determined, only the ones for which polymorphism has been identified in the past. However, for determination of NK ligand positions all of these methods can be used.

Determining the nucleotide sequences can be done with either Sanger sequencing or with next generation sequencing. For both methods the HLA gene of interest is amplified and this amplification comprises at least exons 2 and 3, but can be extended to the full length gene. Subsequent sequencing by the Sanger method might result in an ambiguous allele assignment, because the two alleles were not separated before sequencing and therefore, the cis-trans positions of nucleotides cannot be determined. To circumvent this problem, an allele-group specific amplification can be performed as described previously (88). Alternatively, next generation sequencing also separates the alleles in their processing steps, enabling unambiguous allele assignment as well. Different next generation sequencing methods for HLA class I (HLA-A, -B, -C) have been described, varying from second generation (Illumina, Ion Torrent, Mia Fora), in which small overlapping fragments are sequenced (89–96) to third generation (PacBio), in which a single molecule template is used for sequencing (86, 97), with the notification that for the second generation NGS cis-trans positioning can still be a problem if polymorphism in overlapping fragments is not sufficiently high, resulting in phasing problems and an ambiguous allele assignment. The latest development, often called the fourth generation (Nanopore MinION) is also based on a single molecule, but no nucleotide incorporation is needed, because the nucleotides are identified by passing an electric signal through a nanopore. This latter method is now also validated and implemented for HLA class I high resolution typing (98). By typing to the allele level the KIR ligand epitope is defined.

Since sequencing used to be an expensive and labor intensive method and since NK ligand binding of HLA class I is limited to the amino acid region 77-83, several other methods, focusing only on the polymorphism defining the KIR ligand specificity, were developed in the past. Among them are: (1) a SNP (single nucleotide polymorphism) assay (99), amplifying part of the gene and using fluorescent probes distinguishing between C1 and C2 and between Bw4 and Bw6, the latter one being based on the nucleotide dimorphism at position 319 (amino acid 83), (2) a qPCR Taqman method (100), using HLA-C group 1 and group 2 specific amplification primers in combination with a generic probe for quantification, (3) a PCR-SSP method (101) using sequence specific primers for HLA-C group 1 and group 2 analyzed by gel electrophoresis and (4) a pyrosequencing method (102, 103) based on direct sequencing of the actual ligand epitope and detection by release of pyrophosphate. Furthermore, although the Luminex bead based SSO method, using sequence specific probes bound to fluorescently labeled beads, generates a low resolution typing, it is still possible to determine the HLA ligand presence, because there are probes on the beads that specifically can bind the NK ligand region, both for HLA-B as well as HLA-C. Positivity of these beads implicates that the NK ligand is present.

However, all these methods, that only identify the crucial positions for KIR binding, are not able to detect if the allele present will be really expressed or not. Among both HLA-B and -C (as well as -A) there are now many alleles identified that are not expressed, the so called null alleles. Although their frequencies in the population are very low (104), the impact on NK alloreactivity is huge. Therefore, high resolution typing of HLA-A, -B and -C, according to the standards of the European Federation for Immunogenetics (EFI) taking null alleles into account, would be the definite correct way of determining NK ligand presence.

## Methods to Determine KIR

The KIR genes are comparable to HLA, they also show a high degree of polymorphism, correlated with population groups of specific geographic ancestry, and the more they are studied, the more different alleles are identified (105, 106). However, KIR has two extra layers of variability compared to HLA in that genes can be present or absent and the copy number of genes can vary (107–110). Also for KIR typing different methods are available: methods to determine which genes are present, methods to define how many copies are present and methods to identify the alleles.

Most methods that exist for KIR typing focus on the gene content. Determination of KIR genes present can be performed by PCR-SSO, preferably with Luminex technology so that many samples can be analyzed simultaneously (111), (multiplex) PCR-SSP (112–115), real time PCR (116) or multiplex PCR followed by NGS (117). With these KIR genotyping methods it is also possible to determine the presence of KIR haplotypes A and B, based on the KIR gene presence. The KIR haplotype A has a fixed number of 7 genes (2DL1, 2DL2/3, 2DL4, 2DS4, 3DL1, 3DL2, 3DL3) and 2 pseudogenes (KIR2DP1, KIR3DP1), whereas the KIR haplotypes B vary in KIR gene content, including at least one of the following genes KIR2DL5, 2DS1, 2DS2, 2DS3, 2DS5 and 3DS1 (106, 108, 109, 118).

For defining the number of copies (CNV = copy number of variation) of the different KIR genes different methods have been used, among them quantitative PCR (119), droplet digital PCR (120), multiplex ligation dependent probe amplification (MLPA) (121) and NGS (105, 122). For identifying the KIR alleles, sequencing of the KIR gene is needed. To our knowledge this has only been performed by NGS methodology (105, 122, 123). The Stanford University has developed a method to determine all in one, that means HLA class I KIR ligand typing together with KIR gene presence, copy number and allelic typing. This is achieved by capturing the KIR and HLA class I genes by specific probes, followed by Next Generation Sequencing and a sophisticated bioinformatics pipeline for analysis (105).

As for HLA there is also a database for KIR alleles (https://www.ebi.ac.uk/ipd/kir/) (124) with rules for the assignment and nomenclature of alleles comparable to HLA (125) (**Figure 3**). At present there are 1532 KIR alleles identified, according to this database (vs2.10.0), encoding 668 different proteins, whereas 17 null alleles have been found.

## Non-Classical HLA Class I Molecules as Ligands for NK Cell Receptors in Kidney Transplantation

In contrast to the highly polymorphic classical HLA class I molecules, non-classical HLA class I molecules display a relatively limited polymorphism. HLA-E and HLA-G are the most frequently studied ligands for NK cells and especially their immunosuppressive effects through inhibitory receptors are well characterized. In the following paragraphs the potential impact of the interaction between non classical HLA class I molecules and NK cells expressing receptors that can interact with these molecules on kidney transplantation will be discussed.

### HLA-E as Ligand for NK Cells Expressing NKG2A and/or NKG2C

HLA-E has a gene- and protein structure that is highly comparable to the classical class I molecules but only two main protein variants are known HLA-E*01:01 and HLA-E*01:03 (126, 127). HLA-E can inhibit NK cells and T cells and has been shown to induce regulatory T cells and as a result expression of HLA-E in tissues has been associated with immune suppression in pregnancy and cancer and with viral immune-escape (128). For cell surface expression, binding of a conserved nonamer peptide is required. Due to a single amino acid difference (R107G), the HLA-E*01:03 variant has a higher peptide binding affinity resulting in a higher level of expression on the cell surface (126, 127). Both variants present peptides derived from the leader sequences of HLA-A, -B or -C as well as peptides from viral- and stress proteins (e.g. CMV or Hsp60) (129, 130). The role of this functional dimorphism of HLA-E in solid organ transplantation has been studied in only a very limited number of studies. One study showed a protective role of the HLA-E*01:01/01:01 genotype of the donor for rejection in 107 kidney transplantation donor-recipient pairs (131). In another study, living-donor kidney recipients with HLA-E*01:01/01:01 experienced less BK polyoma virus (BKPyV) reactivation than recipients with other genotypes, and BKPyV-induced nephropathy occurred more frequently in recipients carrying the HLA-E*01:03 allele (132). In both studies, direct effects on NK cell reactivity were not addressed and larger studies are required to draw strong conclusions on the impact of the HLA-E functional dimorphism.

HLA-E can interact with the TCR on CD8 and regulatory T cells and with receptors of the lectin-like family like NKG2A and NKG2C that are expressed on NK cells and some T cell subsets (133). This clearly illustrates that HLA-E can provoke both immune activating as well as inhibitory effects (133). While both NKG2A and NKG2C heterodimerize with CD94, the inhibitory NKG2A family member binds HLA-E with a higher affinity than the activating NKG2C receptor (134). For both receptors, the outcome of receptor ligand interaction depends primarily on the HLA-E expression level as well as on the exact peptide presented in HLA-E (135). Presentation of cellular stress-associated Hsp60 peptides for example does not lead to inhibition of NK cell effector function, while presentation of HLA class I leader peptides or peptides from viral origin does (136, 137). For NKG2C, interaction with HLA-E complexed to an HLA-G derived peptide most potently stimulates NK cells (136, 138). Like the inhibitory KIR family members, NKG2A is involved in licensing of NK cells and NKG2A licensed NK cells can mediate more potent responses against HLA-E negative target cells than their non-licensed hyporesponsive counterparts that do not express KIR or NKG2A (139). Together with the iKIRs, NKG2A is critical in maintaining NK cell tolerance for healthy cells (133). Expression of NKG2C, on the other hand, is largely influenced by viral status of the patients and has been associated with improved anti-viral NK cell responses and long-lived NK cells that have acquired features resembling the adaptive immune response (140, 141).

The functional impact and imprinting that viruses can have on the NK cell compartment is most clearly illustrated by CMV. For a more complete overview on all the studies addressing this topic, we refer to (142), here we will only briefly discuss the impact of CMV on the NK cell receptors interacting with HLA class I because expression levels of both NKG2A and NKG2C are heavily influenced by CMV. One of the first studies providing evidence for this showed that in CMV seronegative individuals, the percentage of NKG2A+ NK cells ranged between 23.5-62.7% while this was between 12.3-75% in CMV positive individuals (143). In the same study, the percentage of NKG2C+ NK cells ranged between 0.1-6% in CMV negative individuals and between 2.5-80% in CMV positive individuals, clearly illustrating the impact of viral imprinting on the NK cell repertoire. Lopez et al. showed that NKG2C^brigth^ NK cells in CMV seropositive blood donors co-expressed CD57 and mostly lacked NKGA and KIR3DL1 and degranulated stronger upon activation by plate bound antibodies (144). A subsequent longitudinal follow up of solid organ transplantation patients showed that NKG2C positive NK cells preferentially expand upon acute CMV infection and that these expanding cells acquire higher levels of NKG2C and co-expression of CD57 (144). Those so called “memory-” or “adaptive” NK cell subsets have also been observed upon CMV reactivation after SCT and are characterized by more potent effector function and a longer lifespan (145, 146). Direct evidence for a CMV-induced imprint of the NK cell compartment was obtained in *in vivo* studies showing that long-lived Ly49H positive NK cells, that were able to mount recall responses, preferentially expanded in mice upon MCMV infection (147). Moreover, *in vitro* studies showed that CMV-infected cells induce proliferation of NKG2C^brigth^ NK cells (148) although the molecular basis has not been fully resolved, interaction with HLA-E presenting CMV derived peptides is one of the main mechanisms suggested to drive the response (149, 150).

HLA-E is expressed by virtually every nucleated cell. Under normal conditions expression levels are low but like the classical HLA class I molecules, HLA-E cell surface expression can be enhanced under inflammatory conditions. Enhanced expression of HLA-E was observed in renal allograft biopsies in patients experiencing acute cellular rejection while this was not observed in patients without any signs of rejections (151). The higher levels of HLA-E were paralleled with increased numbers of CD8 and CD56 positive cells and a higher expression of NKG2C on these effector cells in renal tissue as well as in renal blood vessels. NKG2A was, in this study, almost completely absent on the effector cells suggesting a predominantly activating role for HLA-E in this setting and a role in deterioration of graft function and a higher risk of graft loss (151). Only one study directly evaluated the interaction between HLA-E and NK cells in the solid organ transplantation setting showing that transgene expression of human HLA-E could protect pig endothelial cells in organs like the kidney and the heart against xenogeneic anti-pig cytotoxicity of human NK cells (152). HLA-E can bind peptides derived from several of the viruses known to give complications in the kidney transplantation setting as well as peptides derived from stress proteins. Moreover, HLA-E expression levels on the graft may be influenced by factors from the local microenvironment such as cytokines, DAMPs or PAMPs. Hence, it will be interesting to acquire a better understanding of the role of HLA-E in antiviral immunity after transplantation and rejection by more in depth analysis of HLA-E expression levels, -peptidome and the receptor repertoires of immune cells in the graft. To promote NK cell anti-viral immunity, blockade of NKG2A with HLA-E may be an interesting opportunity to explore, for example with clinically available monoclonal antibodies like monalizumab (153). Though, evidently, this should be tightly balanced to avoid graft rejection that may occur due to lower inhibitory effects of HLA-E.

### HLA-F as Ligand for NK Cells Expressing KIR3DL2/LILRB1 and/or KIR3DS1

The HLA-F gene has a similar structure as the other HLA class I genes, except for the 3’untranslated region (154). Also exon 7 of the HLA-F gene remains untranslated leading to the production of shorter cytoplasmic tail than the other class I molecules (155). HLA-F is expressed intracellular in resting cells (156) and on the cell surface of certain cells including activated lymphocytes (157) and virus infected cells (158). On the cell surface, HLA-F has been found in two different forms; as a heterodimer with β2m and peptides, and as an open conformer without β2m and peptides (159, 160). The different conformations of the HLA-F molecule play an important role in determination of the type of NK cell receptor binding. It has been shown that open conformation binds to inhibitory KIR3DL2 and activating KIR3DS1 (159, 161, 162) while the heterodimer form binds to Ig like transcript (LILRB1 and LILRB2) (160). Although HLA-F has not been studied as intensively as HLA-E and G, increasing body of evidence reveal its clinical relevance in various pathologies including virus infection (163–165), pregnancy (166, 167), autoimmune diseases (168) and cancer (169, 170). However, until now, there are no investigations regarding the role of HLA-F in solid organ transplantation.

### HLA-G as Immune Checkpoint for NK Cells Expressing KIR2DL4 and/or LILRB1

HLA-G is the most frequently studied non-classical HLA class I molecule in the kidney transplantation setting. In contrast to HLA-A,-B,-C and -E, HLA-G is expressed on a restricted set of cells and tissues and mainly in tissues characterized by a tolerogenic- or immune suppressive immune environment (171). Clear examples are the expression of HLA-G on trophoblast cells of the developing fetus during pregnancy or the enhanced expression of HLA-G on tumor or tumor-accessory cells contributing to immune evasion in cancer (172, 173). Also in the solid organ transplantation setting, HLA-G induced tolerance for the allograft has been described: In brief, HLA-G contributes to short- term tolerance by interacting with inhibitory receptors, like LILRB1/2 and KIR2DL4 on immune effector cells. Moreover, it contributes to long-term tolerance *via* the induction of tolerogenic-, IL-10 producing dendritic cells that promote regulatory T cells. In this review, we will focus on the effects of HLA-G on NK cells, the different mechanism of tolerance induction for other immune effector cells have been comprehensively summarized in (174, 175).

Like the other non-classical HLA class I molecules, the HLA-G gene displays only limited polymorphism as compared to HLA-A, -B, or -C. However, an important difference with the other non-classical HLA genes is the frequent occurrence of alternative splicing of the HLA-G gene leading to seven HLA-G isoforms that can be expressed either in a membrane bound- (HLA-G1-4) or in a soluble- (HLA-G5-7) form. Soluble isoforms are the result of alternative splicing of the transmembrane region encoded by exon 5 and can form HLA-G dimers that can signal more potently than monomeric variants (176, 177). The HLA-G1 and HLA-G5 isoform have three extracellular domains of the heavy chain and bind non-covalently to β2M. All the other isoforms express the α1 domain in combination with an α3 domain (HLA-G2 and HLA-G6), with an α2 domain (HLA-G4) or without any additional α domain (HLA-G3 and HLA-G7) (176, 178). Another unique feature is the HLA-G promotor that has a modified regulatory enhancer A (enhA) and lacks interferon-stimulated response elements (ISRE) making it unresponsive to NFκB and IFN-ϒ (179, 180). Consequently, HLA-G expression is not triggered by the typical stimulators of the other HLA class I genes which partly explains its’ tissue restricted expression. HLA-G expression levels are also influenced by multiple SNPs in the promotor region, for example the -725G/T/C polymorphism in the promotor region results in higher HLA-G expression levels for -725G compared to -725C or -725T alleles (181). Given its important role in establishing and maintaining immune tolerance, HLA-G polymorphisms and levels of soluble HLA-G have been frequently determined as surrogate markers for several diseases and pathologies, and multiple studies demonstrated that high levels of membrane bound- or soluble HLA-G were associated with allograft acceptance and less occurrence of acute- or chronic graft rejection summarized in (175, 176).

In addition to the above mentioned mechanisms, HLA-G expression levels can be influenced by genetic variation in the 3’-untranslated region (3’ UTR). Best characterized example, is the 14-base pair insertion or deletion fragment (14-bp INDEL) in exon 8 that, in 14-bp insertion allele variants, leads to the extra deletion of a 92-bp region at the start of exon 8 and enhanced mRNA stability (182). Another example is the C/G SNP on position +3142 that influences HLA-G targeting micro-RNA binding and, by doing so, mRNA stability (175). One of these micro-RNAs, miR365, is enhanced under hypoxic conditions (183) and it may be relevant to study its contribution to ischemia-reperfusion damage in the kidney transplantation setting. Moreover, the presence of the +3142CC genotype in kidney transplant recipients (n=178), as well as higher soluble HLA-G levels, have been associated with higher susceptibility to CMV infection (184). A third example of the influence of genetic variation on HLA-G expression, is an A/G SNP on position +3187 that has been related to reduced HLA-G expression in +3187A alleles due to its proximity to the AU-rich motive (175). 3’ UTR variation has been associated with multiple pathologies, including allograft rejection, where protective effects against rejection have been described for the 14-bp ins/ins and +3142 GG homozygous genotypes of the donor kidney (185). The linkage disequilibrium of the 14-bp ins/del and the +3187- and +3142 SNPs complicates analysis of the contribution of the individual regions to transplantation outcome. Hence, in a recent study, 3’ UTR haplotypes were determined based upon fourteen 3’ UTR SNPs (between +2960 and +3227) and the 14-bp ins/del (186). Subsequent deduction of the SNPs responsible for the observed effects, led to the identification of a +3003C SNP variant that, when present in donor as well as in recipient, was associated with BKPyV/PyVAN (Polyomavirus-associated nephropathy) occurrence and protection against antibody mediated rejection, while, the +3196G variant was associated with enhanced graft rejection (186).

Receptors for HLA-G are expressed on numerous immune cells including NK cells. LILRB1 (alias LIR1 or ILT2) and KIR2DL4 are the most well described receptors for NK cells. LILRB1 interacts with β2M-associated HLA-G molecules and exclusively acts as an inhibitory receptor for NK cells (187). KIR2DL4, one of the framework KIR genes, is predominantly expressed by decidual NK cells and both inhibitory- and activating-effects have been described upon interaction with HLA-G (188–191). Despite the numerous studies suggesting an important role for HLA-G to maintain allograft tolerance, the direct impact of HLA-G on NK cells in the kidney transplant setting or associations between HLA-G binding NK cell receptors is almost completely lacking. In a cohort of 81 healthy individuals *vs* 82 renal transplant recipients, a SNP in LILRB1 (rs1061680) has been shown to associate with increased carotid intimal media thickness (192) but additional comprehensive studies evaluating LILRB1 expression or genetic variation in kidney transplantation are lacking. By comparing 90 patients with a functional renal allograft and 40 patients rejecting their transplant, Ajith et al. confirmed the protective effect of high levels of the soluble HLA-G dimers (193). They also studied the underlying mechanism by genomic- and cellular analysis of patient derived T cells and in LILRB1 transgenic mouse models. This revealed that the HLA-G soluble dimers reduced the level of granzyme B in CD8 T cells in a LILRB1 dependent manner hence making them less cytotoxic. Although they did not address the role of NK cells in detail, a comparable mechanism may be relevant for the effect of soluble HLA-G on LILRB1 expressing NK cells as CD8^+^ T cells, since NK cells use comparable mechanisms for target cell elimination.

## Non-Classical HLA Class I Determination

Since typing of the classical class I molecules has been mandatory for transplantation purposes, many different DNA typing techniques and commercial kits are available. In contrast, typing of the non-classical HLA class I, HLA-E, -F and G genes, has been rather fragmentary.

For HLA-E, the typing method has long been limited to resolve the dimorphic amino acid at position 107 (R or G) by either PCR-SSP (PCR with Sequence-Specific Primers (194–197), PCR-SSO (PCR-Sequence Specific Oligo Probes (198), PCR-RFLP (PCR Restriction Fragment Length Polymorphism) alone (199) or in combination with ARMS (Amplification Refractory Mutation System) (200), PCR-SSCP (PCR-single strand conformation polymorphism) (201), Taqman assay (202) or sequence based typing of a limited part of the HLA-E gene either by Sanger sequencing (203–207) or recently also by NGS with Illumina (208). In this latter study HLA-E was typed for over 2.5 million potential stem cell donors worldwide and although only a limited 535 bp amplicon (including last part of exon 2, intron 2 and first part of exon 3) was sequenced, it has caused an explosion of new HLA-E alleles (209). Also full length sequencing of HLA-E was developed using both Sanger sequencing (210, 211) and NGS (212–216). These full length strategies have also revealed new alleles, including alleles with polymorphism present outside of the peptide binding groove. Although at present (IPD-IMGT/HLA database version 3.44.0) 271 different HLA-E alleles and 110 different HLA-E protein molecules have been recognized, the two major proteins HLA-E*01:01P (R107) and E*01:03P (G107) account for >99% of the population. In fact, in a huge study with > 2.5 million individuals typed for HLA-E, only in 0.05% another allele (01:05, 01:07 or new) was detected. Among the 110 different HLA-E protein molecules, there are only 2 that have an amino acid at position 107 that is different than the HLA-E*01:01/*01:03 main protein variants, namely HLA-E*01:48 (K107) and E*01:88 (S107). While the R107G SNP impacts HLA-E expression levels (126), the functional impact of the K107 and S107 change is not clear. Also the functional impact of amino acid differences at other positions, if any, is unclear. The 7 null alleles are all due to a single nucleotide difference, changing an amino acid coding codon to a stop codon (TAA, TGA or TAG) and all of them are located between positions 84 and 113, and therefore can easily be recognized if only part of the gene is sequenced.

HLA-F is the least polymorphic of the classical and non-classical HLA class I genes (A, B, C, E, F, G). In the present IPD-IMGT/HLA database (3.44.0) a total of 45 different alleles has been identified, but they only encode 6 different proteins. The amino acid differences are located at the start or the end of the protein, leaving the middle part (amino acids 51 – 250) identical for all hitherto known HLA-F alleles. There is also no null allele yet identified, implicating an important role for this conserved protein. Since there is only limited polymorphism identified, the methods to type for HLA-F have often been limited to the known polymorphism or to a fraction of the gene, enabling only identification of the known alleles. The method dealing with the known polymorphism that has been used for HLA-F typing is PCR-SSP (217), whereas in several studies part of the gene has been amplified and sequenced by Sanger sequencing (168, 204, 218). In more recent studies NGS of the whole gene has been used for characterizing the HLA-F gene in several different populations (212, 219, 220) or as part of identification of all HLA genes in a multiplex set up (215).

Comparable to HLA-E and HLA-F also HLA-G exhibits few polymorphic sites along the sequence (221), although it might have attracted more attention because of its role in materno-fetal tolerance and the different splicing forms that have been identified, resulting in different soluble and membrane bound isoforms. At present there are 82 different HLA-G alleles in the IPD-IMGT/HLA database (3.44.0), encoding 22 different proteins, whereas 4 null alleles have been identified. Due to a rather conserved molecule more attention has been paid to analysis of the promotor region (5’ upstream regulatory region, 5’ URR) and the 3’ UTR region that are thought to play a role in the expression levels of HLA-G and thus influence its immuno-tolerogenic (or immunomodulatory) properties (173, 222–226). Especially the 14 bp insertion/deletion that was identified in the 3’ UTR region and correlated with mRNA stability and thus expression levels has been intensively studied by different methods, ranging from real time TaqMan PCR (227), amplification of part of the 3’ UTR region followed by size discrimination analyzed by gel electrophoresis (228–230) to sequencing of this 3’ UTR region (231–233). Not only 5’ URR and 3’ UTR are important, also the amino acid differences in the alpha2 domain have been found to influence the peptide binding repertoire resulting in functional differences between different HLA-G subtypes (234). Typing to identify the HLA-G alleles has mainly been performed in the framework of population and evolutionary studies and studies on reproduction, infection and disease associations, often limited to sequencing of exons 2-4 (230, 235, 236), sometimes combined with identification of the 14 bp insertion/deletion in the 3’ UTR region (237–239). Recently, also NGS has been used to identify the HLA-G alleles present (212, 215, 240–242). A high linkage disequilibrium was found between the HLA-G allele type and the polymorphism in the 3’ UTR region (241).

## Short Discussion/Conclusion

In the present review, we discussed the role of classical and nonclassical HLA class I molecules as immune checkpoints for NK cells and the relevance for two important determinants of kidney transplantation outcome: graft survival/rejection and viral infection. While several studies showed that expression levels- and soluble variants of non-classical HLA-E and -G are associated with rejection, data on the direct effects on NK cells and the contribution of NK cell receptors with specificity for non-classical HLA class I molecules in the kidney transplantation setting is rather limited. The contribution of KIR genes and iKIR-HLA class I matching status have been studied much more frequently. Multiple studies support the hypothesis that incompatibility between iKIRs in the recipient and HLA ligands in the graft may be detrimental for allograft survival. From the stem cell transplantation setting, it is becoming more and more clear that the impact of KIR-ligand matching *vs.* mismatching is greatly influenced by the exact transplantation protocol and beneficial effects of KIR-ligand mismatching were primarily seen in the severely T cell depleted setting (243). This may be explained by a reduced post-transplant pharmacological GvHD prophylaxis in this setting as such therapies have been shown to obscure the NK cells effects (244). Moreover, the exact model used to evaluate matching status could also influence the outcome as, in some studies, conclusions were drawn based on analysis of the presence *vs.* absence of HLA epitopes while in other studies this was complemented with data on the genotypic- or even phenotypic presence of the corresponding *KIR* genes. Since HLA and KIR are encoded on different chromosomes and KIR null alleles exist it would be relevant to address the relevance of KIR-ligand mismatching in larger cohorts using different models to determine matching status. In addition, functional studies could be used to further dissect the importance of KIR-ligand matching and this could also be related to the different immunosuppressive regimen. Most pronounced were the effects observed for the *KIR* gene repertoire and KIR haplotype and they were identified by various studies as important determinants of the immune response against the certain serotype of viruses. This illustrates that assessing genetic profiles of NK cell receptor with specificity for HLA class I may be useful to improve transplantation outcome. Given the dual role of NK cells in transplantation, it would be relevant to perform follow up studies to further evaluate the predictive value of those genetic profiles for combined endpoints, i.e. taking into account the occurrence of both graft rejection and infectious disease. The advancement in molecular typing methods for both HLA and NK cell receptors provides better discrimination of subtype of HLA alleles and specific types of KIR receptors and enables this type of analysis. Combined with functional- and spatial analysis of immune cell infiltration and -function and the identification of additional ligand-specificity for aKIR, this will facilitate the deeper understanding of the role of NK cell immune checkpoints in kidney transplantation, which may guide the exploitation of targeting NK cells for therapeutic benefits.

## Author Contributions

BD, TO, MG, CV, and LW wrote sections of the manuscript and reviewed it. All authors contributed to the article and approved the submitted version.

## Conflict of Interest

The authors declare that the research was conducted in the absence of any commercial or financial relationships that could be construed as a potential conflict of interest.
